# CD103 Deficiency Prevents Graft-versus-Host Disease but Spares Graft-versus-Tumor Effects Mediated by Alloreactive CD8 T Cells

**DOI:** 10.1371/journal.pone.0021968

**Published:** 2011-07-14

**Authors:** Kechang Liu, Bryan A. Anthony, Martha M. Yearsly, Mehdi Hamadani, Alice Gaughan, Jiao-Jing Wang, Steven M. Devine, Gregg A. Hadley

**Affiliations:** 1 Department of Surgery, The Ohio State University, Columbus, Ohio, United States of America; 2 Department of Microbiology and Immunology, University of Maryland Medical School, Baltimore, Maryland, United States of America; 3 Department of Pathology, The Ohio State University, Columbus, Ohio, United States of America; 4 Department of Hematology and Oncology, West Virginia University, Morgantown, West Virginia, United States of America; 5 Department of Hematology and Oncology, Arthur G. James Comprehensive Cancer, The Ohio State University, Columbus, Ohio, United States of America; Saint Louis University, United States of America

## Abstract

**Background:**

Graft-versus-host disease (GVHD) remains the main barrier to broader application of allogeneic hematopoietic stem cell transplantation (alloSCT) as a curative therapy for host malignancy. GVHD is mediated by allogeneic T cells directed against histocompatibility antigens expressed by host tissues. Based on previous studies, we postulated that the integrin CD103 is required for CD8-mediated GVHD, but not for graft-versus-tumor effects (GVT).

**Methodology/Principal Findings:**

We herein provide evidence in support of this hypothesis. To circumvent the potentially confounding influence of donor CD4 T cells, we developed an alloSCT model in which GVHD mortality is mediated by purified CD8 T cells. In this model, host-reactive CD8 T cells receive CD4 T cell help at the time of initial activation but not in the effector phase in which mature CD8 T effectors migrate into host tissues. We show that donor CD8 T cells from wild-type BALB/c mice primed to host alloantigens induce GVHD pathology and eliminate tumors of host origin in the absence of host CD4 T cells. Importantly, CD103 deficiency dramatically attenuated GVHD mortality, but had no detectable impact on the capacity to eliminate a tumor line of host origin. We provide evidence that CD103 is required for accumulation of donor CD8 T cells in the host intestinal epithelium but not in the tumor or host lymphoid compartments. Consistent with these data, CD103 was preferentially expressed by CD8 T cells infiltrating the host intestinal epithelium but not by those infiltrating the tumor, lamina propria, or lymphoid compartments. We further demonstrate that CD103 expression is not required for classic CD8 effector activities including cytokine production and cytotoxicity.

**Conclusions/Significance:**

These data indicate that CD103 deficiency inhibits GVHD pathology while sparing anti-tumor effects mediated by CD8 T cells, identifying CD103 blockade as an improved strategy for GVHD prophylaxis.

## Introduction

The potential of allogeneic hematopoietic stem cell transplantation (alloSCT) to eliminate host malignancy is limited due to graft-versus-host disease (GVHD) mediated by donor T cells[1]. Although multiple means exist to neutralize donor-reactive T cells, such strategies also inhibit anti-tumor effects (GVT), leaving the host vulnerable to disease relapse [2]. CD8 T cells are important mediators of acute GVHD and GVT effects following alloSCT due to their capacity to cross-react at high frequency with polymorphic variants of MHC class I molecules [3], and recognize polymorphic peptides derived from non-MHC proteins (i.e., minor H antigens) in the context of self MHC class I molecules [4]. Thus, even MHC-matched transplants elicit potent immune responses mediated by donor CD8 T cells. Moreover, CD8 T effectors elicited in response to host alloantigens possess diverse effector pathways for destruction of host cells. Ubiquitous expression of MHC I molecules assures that all host cell-types are potentially susceptible to CD8-mediated injury. The relevance of these data to clinical events is supported by studies showing that depletion of CD8 cells from the alloSCT inoculum attenuates GVHD episodes [5], [6] in the human system.

We have previously reported that the expression of the integrin CD103 by CD8 T effector populations is required for development of intestinal GVHD pathology and associated mortality following alloSCT [7]. The known ligand for CD103 (E-cadherin) is generally lost by epithelial tumors during transition to invasive carcinoma [8], yet most tumor cells retain high level expression of LFA-1 ligands, such as ICAM-1[9]. Le Floc'h et al. [10] have reported that tumor-reactive CTL clones use LFA-1-dependent interactions for tumor lysis when CD103/E-cadherin interactions are not available. These data raised the possibility that CD103 expression is required for GVHD pathology but is dispensable for effective anti-tumor immunity mediated by donor CD8 T cells.

The goal of the present study was to test the hypothesis that CD103 deficiency can prevent GVHD pathology without compromising tumor immunity mediated by alloreactive CD8 T cells. We herein provide evidence in support of this hypothesis, and document that this reflects a requirement for CD103 in accumulation of CD8 T cells in epithelial but not non-epithelial host compartments. That these data provide novel insight into more effective strategies for GVHD prophylaxis is discussed.

## Results

### CD103 deficiency attenuates intestinal GVHD mediated by donor CD8 T cells

To assess the impact of CD103 on GVHD and GVT effects mediated by donor CD8 T cells, we used an MHC-mismatched model (BALB/c-to-A/J, disparate at H-2K^k^, H-2A^k^, and H-2E^k^) to take advantage of the high frequency of CD8 T cells directed to mismatched MHC I alloantigens [3]. To exclude confounding pathology mediated by donor CD4 T cells, CD8 T cells from BALB/c-WT (WT) or BALB/c-CD103 KO mice were purified and adoptively transferred into lethally irradiated A/J mice. Donor mice were primed to host alloantigens prior to transfer to circumvent the well documented requirement for CD4 T cell help in eliciting CD8-dependent GVHD [11]. Thus, host-reactive CD8 T cells in this model received CD4 T cell help at the time of initial activation but not in the effector phase in which mature CD8 T effectors migrate into host tissues.

**Figure 1:**
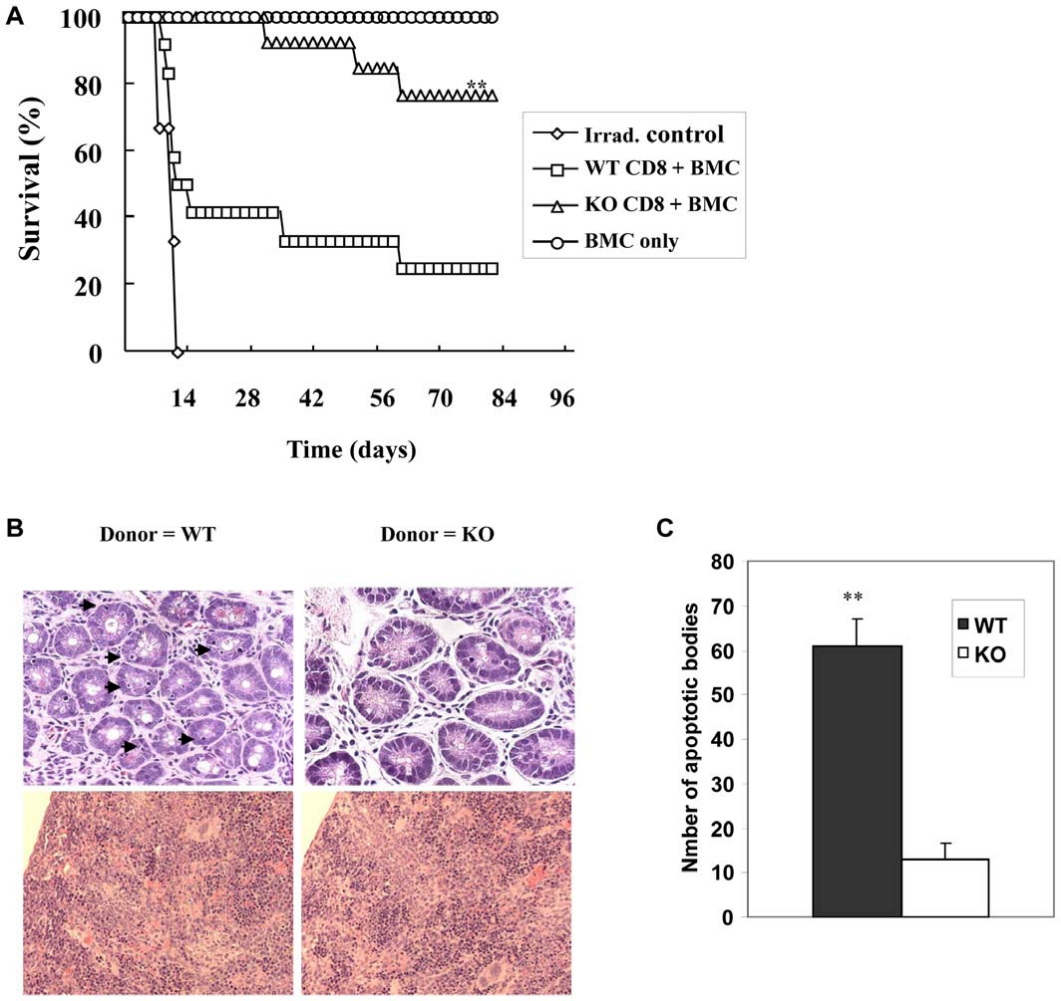
CD103 deficiency attenuates intestinal GVHD mediated by donor CD8 T cells. CD8 T cells primed to A/J alloantigens from either BALB/c-WT (WT) or BALB/c-CD103 KO donors were adoptively transferred into lethally irradiated A/J recipient mice in combination with WT BMC. A. Data shown are survival rates of irradiation control mice (diamonds), recipients of WT BMC plus purified CD8 T cells from either WT (WT CD8, squares, n = 12) or KO donors (KO CD8, triangles, n = 13), and recipients of BMC only (circles, n = 3). These data derive from two separate experiments with similar results. **p-value = 0.003, compared to the survival of hosts receiving WT CD8 T cells (log-rank test). B. H&E sections of the host intestine (upper panel) and spleen (lower panel) taken from recipients of WT or KO CD8 T cells. Arrows indicate apoptotic bodies. C. Mean number of apoptotic bodies per hundred intestinal villi ± SEM in recipients of CD8 T cells from WT donors (WT, closed bar, n = 3) or KO donors (KO, open bar, n = 3). **p<0.01.

As shown in Fig. 1A, purified CD8 T cells from primed WT donors (<1% CD4 T cells) induced GVHD onset as early as day 6 post transfer resulting in 75% (9/12) GVHD mortality within 90 days. In contrast, purified CD8 T cells from KO donors elicited milder or undetectable GVHD resulting in a significantly lower incidence of GVHD mortality (23%; n = 3/13, log-rank p-value = 0.003). Recipients of WT CD8 T cells (WT recipients) also experienced greater weight loss compared to recipients of KO CD8 T cells (data not shown).

Recipient mice were sacrificed at day 12 post-transfer for histologic analyses, a time at which more than half of the recipients of WT CD8 T cells had died of GVHD (Fig. 1A). At this time point, approximately 40% of WT CD8 T cells infiltrating the host intestinal epithelium but not the host spleen expressed significant levels of CD103 (Figure S1). As shown in Figure 1C, significantly higher numbers of apoptotic bodies were present in the intestinal epithelium of recipients of WT CD8 T cells (61±6.0/100 villi) compared to those receiving CD103 KO CD8 T cells (13±3.5/100 villi)(mean ± SEM). Note the absence of apparent differences between WT and CD103 KO cells in pathology elicited in the host spleen (Figure 1B), despite the presence of obvious injury at this site. These data are consistent with a role for CD103 in promoting injury to the host intestinal epithelium mediated by CD8 T cells.

### CD103 is not required for effective clearance of solid tumor by donor CD8 T cells

**Figure 2:**
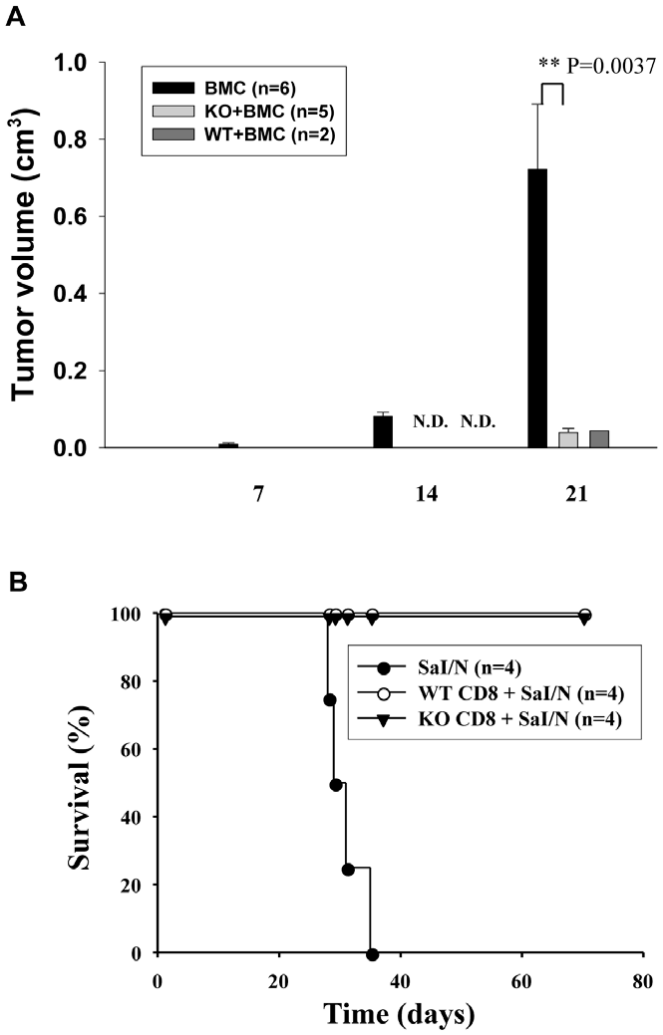
CD103 is not required for effective clearance of solid tumor by donor CD8 T cells. A. Lethally irradiated A/J mice were inoculated with SaI/N fibrosarcoma (H-2^a^) plus BALB/c-WT (WT) either alone (BMC only, n = 6) or in combination with purified CD8 T cells from primed WT (n = 8) or BALB/c-CD103 KO donors (n = 8). Data shown are the mean volumes of solid tumor (cm^3^) measured at the indicated time points ± SEM. N.D. indicates that no tumor was detected. This is a combination of two separate experiments with similar results. Since some experimental mice died early of GVHD, data of 6, 5 or 2 recipient mice of each respective group are shown in last time point. B. Sal/N fibrosarcoma cells (1.0×10^6^) were transferred into BALB.scid mice alone (closed circles; n = 4) or together with primed CD8 T cells (1.0×10^7^) from either WT (triangles; n = 4) or KO donors (open circles; n = 4). Data show mortality of recipient mice. Surviving mice were sacrificed at day 70 after adoptive transfer.

To determine whether CD103 deficiency compromises GVT effects mediated by CD8 T cells in this model, primed CD8 T cells from WT or KO donors were transferred into lethally-irradiated A/J mice together with the A/J background fibrosarcoma, SaI/N. A control group of A/J mice received BMC and SaI/N cells without donor CD8 T cells. As shown in Fig. 2A, tumor growth was detected in all mice transferred with BMC alone within 2 weeks with the majority (5/6) exhibiting massive tumor growth by day 21 post-transfer. In marked contrast, the majority of recipients of WT CD8 T cells (6/8) died of GVHD by day 14 before any detectable tumor growth as compared to 2/8 recipients of KO CD8 T cells (data not shown). Fig. 2A shows that WT and KO CD8 T cells both effectively inhibited tumor growth as reflected by a nearly 20-fold reduction in tumor volume compared to mice receiving donor BMC without CD8 T cells at day 21 post transfer. These data suggested that CD103 expression is not required for GVT effects mediated by donor CD8 T cells.

Given that the majority of recipients of WT CD8 T cells died from GVHD pathology before detection of tumor growth, we could not directly compare GVT effects mediated by WT vs. KO CD8 T cells in this model. To circumvent this problem, we transferred primed CD8 T cells from either WT or KO donors into syngeneic BALB.scid mice together with SaI/N tumor cells. Thus, the hosts are syngeneic to the donor T cells in this model, allowing an evaluation of GVT effects mediated by alloreactive CD8 T cells without the confounding occurrence of GVHD pathology. As shown in Fig. 2B, all mice receiving tumor alone died within 35 days, whereas recipients of CD8 T cells from either WT or KO donors survived for >70 days without detection of tumor growth. Thus, these data indicated that CD103 deficiency does not significantly compromise GVT effects mediated by donor CD8 T cells in this model.

### CD103 expression is not required for accumulation of donor CD8 T effectors in the tumor

**Figure 3:**
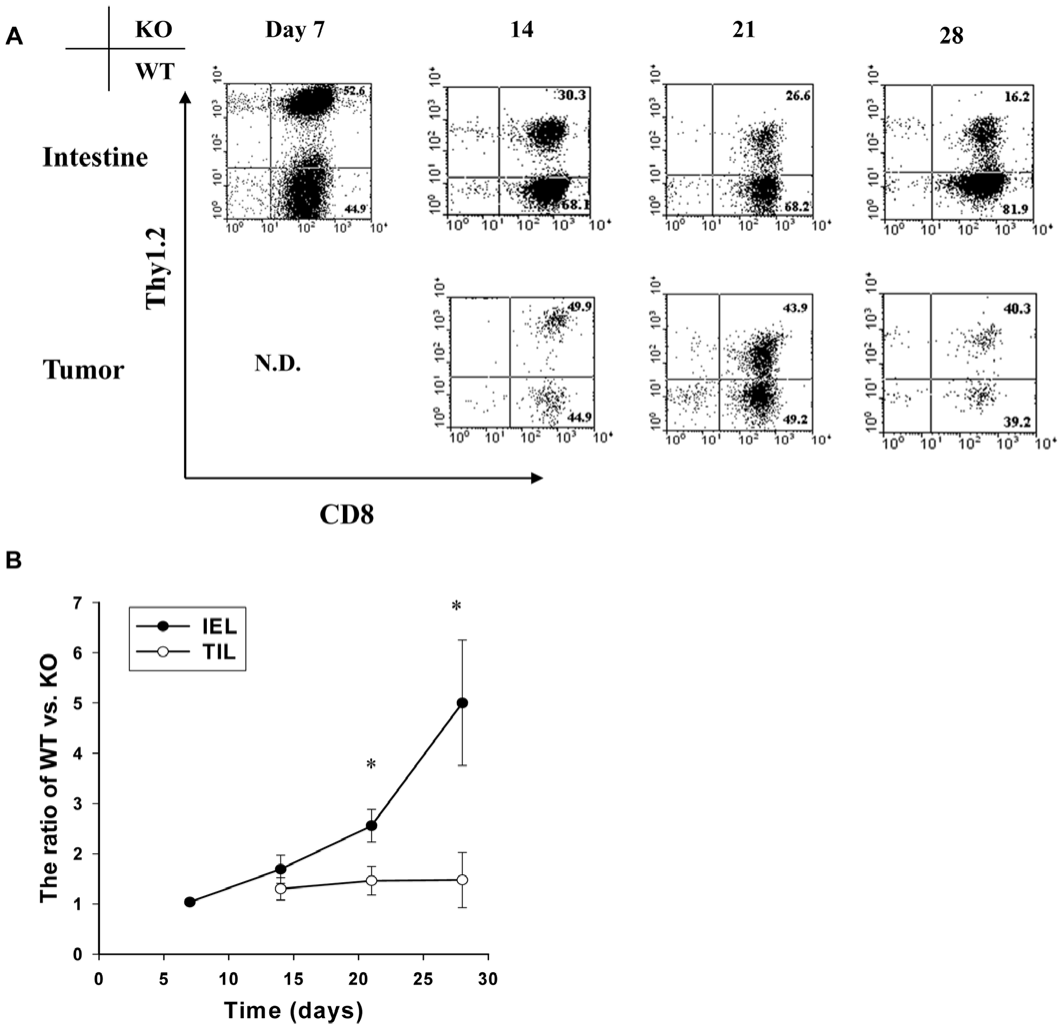
CD103 expression is not required for accumulation of donor CD8 T effectors at the site of tumor growth. Equal numbers of CD8 T cells from BALB/c-WT (WT, Thy1.1^+^) and BALB/c-CD103 KO, (KO, Thy1.1^+^Thy1.2^+^) donors were adoptively transferred into a group of lethally irradiated A/J mice together with BALB/c-WT BMC plus SaI/N tumor cells. Recipient mice were sacrificed at days 7, 14, 21 and 28. Lymphocytes isolated from the host intestinal epithelium and the tumor were subjected to flow cytometric analyses. A. Representative dot plots of Thy1.2 expression by gated CD8 T cells in the two compartments at the indicated time points. B. Mean ratios ± SEM (n = 5) of WT versus KO CD8 T cells in the intestinal epithelim (IEL, solid circles) and tumor (TIL, open circles). *p<0.05 compared with TIL.

**Figure 4:**
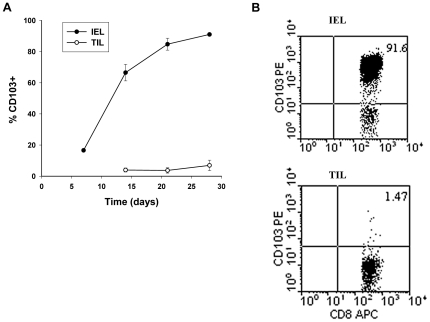
CD103 is preferentially expressed by CD8 T cells in the intestinal epithelium as compared to the tumor. Equal numbers of CD8 T cells from BALB/c-WT (WT, Thy1.1^+^) and BALB/c-CD103 KO (KO, Thy1.1^+^Thy1.2^+^) donors were adoptively transferred into a group of lethally irradiated A/J mice together with BALB/c-WT BMC plus SaI/N tumor cells. Recipient mice were sacrificed at days 7, 14, 21 and 28. Lymphocytes isolated from the indicated host compartments were then subjected to flow cytometric analyses. A. Mean percentage ± SEM (n = 5) of CD8 T cells in the intestinal epithelium (IEL closed circles) and the tumor (open circles) that expressed significant levels of CD103 at the indicated time points. B. Representative dotplot of CD103 expression by gated CD8+ cells in the intestinal epithelium (IEL) and tumor (TIL).

**Figure 5:**
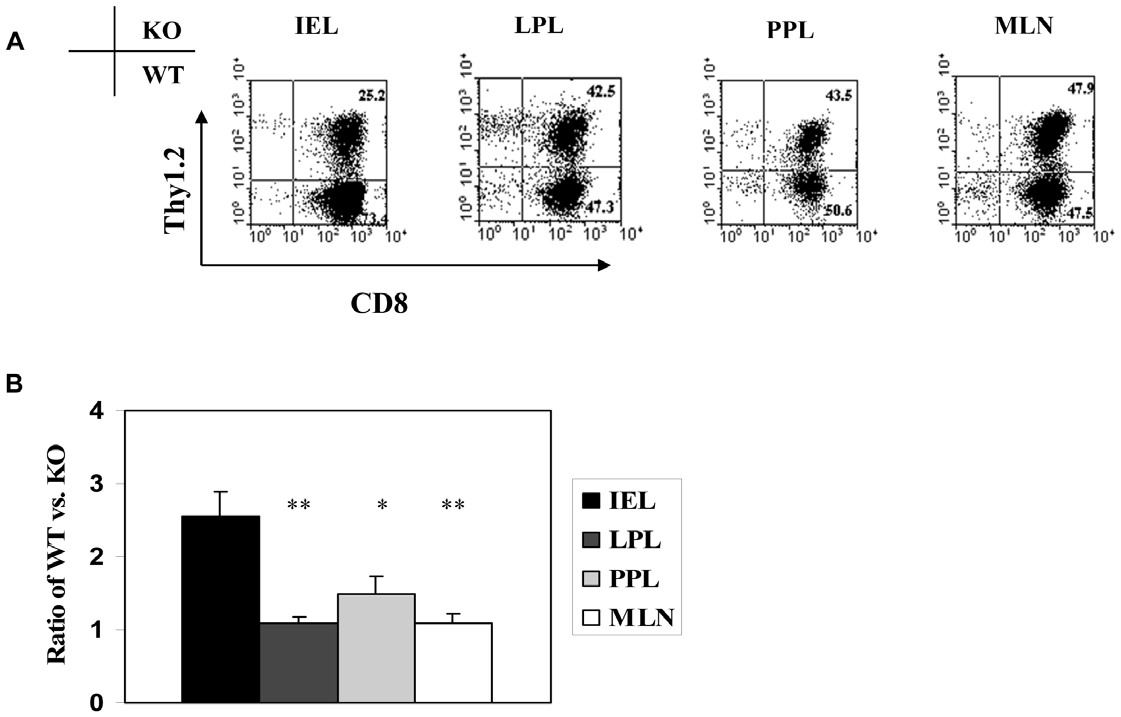
CD103 is not required for accumulation of CD8 T cells in the lamina propria or mucosal lymphoid compartments during GVHD. Equal numbers of CD8 T cells from BALB/c-WT (WT, Thy1.1^+^) and BALB/c-CD103 KO (KO, Thy1.1^+^Thy1.2^+^) donors were adoptively transferred into a group of lethally irradiated A/J mice together with BALB/c-WT BMC plus SaI/N tumor cells. Recipient mice were sacrificed at day 21. Lymphocytes isolated from the indicated host compartments were subjected to flow cytometric analyses. A. Representative dot plots of WT and KO CD8 T cells in the host intestinal epithelium (IEL), lamina propria (LPL), Peyer's patch (PPL), and mesenteric lymph node (MLN) at d21 post alloSCT. B. Mean ratios ± SEM (n = 5) of WT to KO CD8 T cells in the indicated compartments. *p<0.05 compared with IEL; **p-value<0.01 compared with IEL.

The SaI/N tumor line –similar to most human tumors [8] - does not express detectable levels of E-cadherin (data not shown). We therefore postulated that separation of GVHD and GVT effects by CD103 deficiency reflects a lack of requirement for CD103 expression for efficient accumulation of donor CD8 T cells in the tumor and other non-epithelial compartments. To test this hypothesis, an equal mixture of CD8 T cells from WT (Thy1.1) and KO (Thy1.2) donors were transferred in combination with WT BMCs (Thy1.1) into lethally-irradiated A/J hosts together with SaI/N tumor cells. Note that the donors were marked with Thy1 alleles to facilitate identification of the WT or KO origin of CD8 T cells, and that sublethal doses of CD8 T cells were transferred in these experiments to extend host survival. Recipient mice were sacrificed at days 7, 14, 21 and 28, and lymphocytes isolated from host tissues were subjected to flow cytometry. Consistent with our prior study [7], WT CD8 T cells rapidly outnumbered KO CD8 T cells in the host intestinal epithelium by the third week post transfer (Fig. 3A). In contrast, comparable numbers of WT and KO CD8 T cells accumulated in the tumor (Fig. 3A). Fig. 4A shows that CD103 expression by WT CD8 T cells infiltrating the host intestinal epithelium progressively increased from 66% at day 14 to 91% at day 28 post adoptive transfer. In contrast, tumor-infiltrating CD8 T cells of WT origin expressed negligible levels of CD103 at all time points (Fig. 4B), consistent with a lack of involvement of the CD103 pathway in tumor elimination by donor CD8 T cells. Fig. 5 shows preferential accumulation of WT CD8 T cells in the host intestinal epithelium but not in a variety of non-epithelial compartments including the LP, PP, and MLN, despite high level CD103 expression by WT CD8 T cells in the latter compartments (data not shown). These data indicate that CD103 deficiency prevents accumulation of donor CD8 T cells in host epithelial compartments without compromising their capacity to accumulate in non-epithelial compartments, which includes both lymphoid tissues and the tumor itself.

### CD8 T effector function is not attenuated by CD103 deficiency

**Figure 6:**
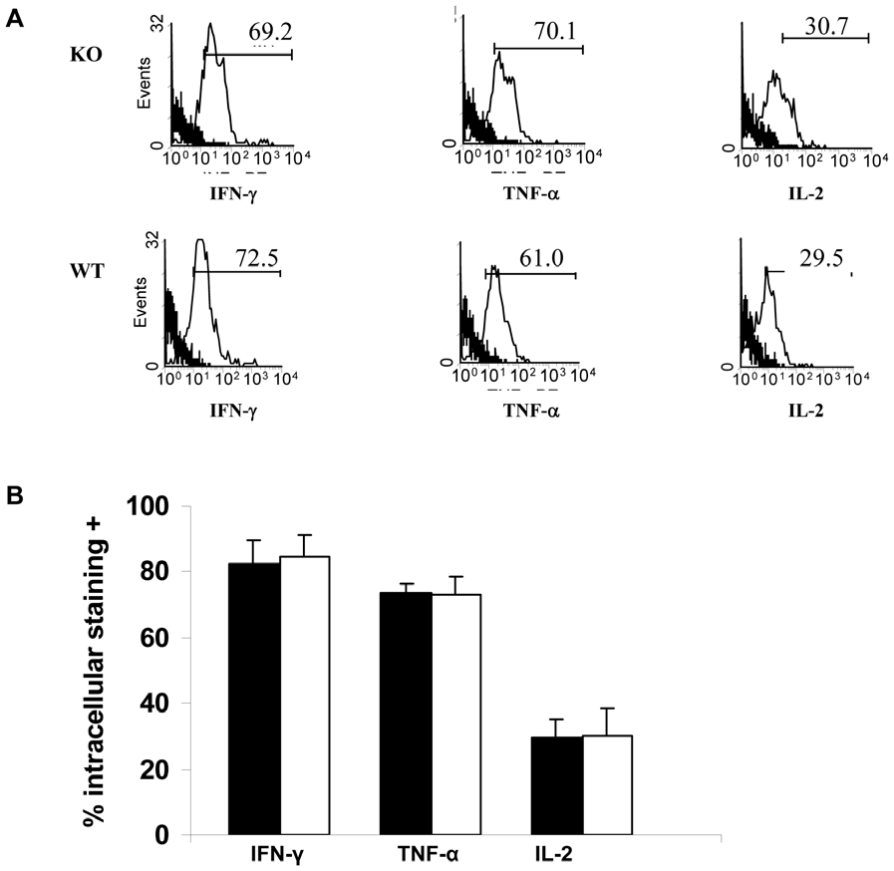
CD103 expression is not required for cytokine production by gut infiltrating CD8 T cells. Equal numbers of CD8 T cells from BALB/c-WT (WT, Thy1.1+Thy1.2-) and BALB/c-CD103 KO donors (KO, Thy1.1^+^Thy1.2^+^) were adoptively transferred into a group (n = 3) of lethally irradiated A/J mice together with WT BMC plus SaI/N tumor cells. Lymphocytes were isolated from the host intestinal epithelium at day 7 post-transplant and subjected to intracellular staining for INFγ, TNFα and IL-2. A. Representative histograms of cytokine staining by gated CD8+ lymphocytes of either WT or KO origin (thin line) vs. isotype control staining (heavy line). B. Mean production (±SEM) of the indicated cytokines by gated WT (n = 3, solid bars) or KO CD8 T cells (n = 3, open bars). Data represent two independent experiments with similar results.

Cytokines produced by CD8 T cells during GVHD responses - particularly IFN-γ, IL-2 and TNF-α - likely play critical roles in promoting GVT effects (1). As shown in Fig. 6, CD8 T cells infiltrating the host intestinal epithelium produced copious amounts of the three cytokines at days 7, independently of their capacity to express CD103. As shown in Figure S2, primed CD8 T cells from KO donors were just as effective as those from primed WT donors in eliminating cells of host origin in vivo. Thus, these data indicate that CD103 expression by CD8 T cells is not required for acquisition of classic effector function by CD8 T cells activated during the course of GVHD.

## Discussion

The salient finding of the present study is that CD103 deficiency prevents GVHD mortality mediated by donor CD8 T cells without compromising their capacity to eliminate tumors of host origin. We have previously documented a key role for CD103 in promoting intestinal GVHD pathology mediated by CD8 T cells [7]. The present data extend the prior studies by showing that CD103 blockade does not abrogate the capacity of donor CD8 T cells to eliminate allogeneic tumors. These data identify the CD103 pathway as being critical to GVHD pathology, yet dispensable for host defense against malignancy. It will now be important to extend these findings to spontaneously developing tumors in the human system. As the most common application of alloSCT is to treat hematopoietic malignancy[1], it will be particularly important to confirm relevance to anti-leukemia effects.

We have previously documented a key role for TGFβ in accumulation of donor CD8 T cells within the host intestinal epithelium during GVHD [7]. The present findings document that CD103 expression is not required for efficient circulation of CD8 T cells through host lymphoid tissues and other non-epithelial sites including the tumor itself (Fig.5). These data are consistent with a model in which host-reactive CD8 T cells entering host epithelial compartments following alloSCT are induced to express CD103 by bioactive TGFβ, a cytokine known to promote CD103 expression by CD8 T cells[12] and which is ubiquitous at GVHD target sites such as the gut epithelium [13]. In this model, CD103 expression promotes accumulation of donor CD8 T cells in host tissues via interaction with E-cadherin, which is highly and specifically expressed by cells comprising host epithelial compartments [14] Our data indicate that CD8 T cells unable to express CD103 remain capable of exerting anti-tumor effects – likely through LFA-1-dependent mechanisms[10] - hence providing a mechanistic basis for the separation of GVHD and GVT effects by CD103 disruption.

That some recipients (∼25%) of KO CD8 T cells succumb to GVHD mortality (Fig. 1) is consistent with our data showing that CD103-deficient CD8 T cells retain the capacity to accumulate in non-epithelial compartments of the host independently of CD103 expression. These data are in accord with observations that tumor-reactive CTL clones utilize LFA-1-dependent interactions as an alternative pathway to promote directed release of cytotoxic granules in lieu of CD103/E-cedherin interactions [10], and migrate to cognate tumor independently of CD103 expression [15].

CD8 T cells have great potential as a means to cure human malignancy but this approach is currently limited by the propensity of these cells to mediate GVHD pathology. Indeed, both alloSCT [5], [6] and delayed leukocyte infusion [16] have the capacity to cure malignancy but both are limited by concomitant GVHD pathology. Multiple means exist to neutralize CD8 T cells to prevent GVHD pathology but current approaches have the potential to abrogate critical anti-tumor responses, and thus are likely ineffectual in the long term. The present data provide proof-of principle that CD103 blockade can prevent GVHD mortality mediated by donor CD8 T cells without compromising GVT effects, thus pointing to CD103 blockade as an inherently superior means of GVHD prophylaxis. We have documented that reagents that deplete CD103-expressing cells in vivo do not significantly compromise T reg function [17], so this approach appears unlikely to interfere with physiologic mechanisms for suppressing tumor growth.

In summary, the present findings indicate that CD103 deficiency attenuates CD8-dependent GVHD pathology while preserving beneficial GVT effects, and shed light on the underlying mechanisms. It will now be important to determine if CD103 depleting agents [17] provide a viable strategy for separating GVHD and GVT effects in WT hosts.

## Materials and Methods

### Mice

Mice of strains C57BL/6 (B6), A/J, BALB/cJ (WT), C.129S2-*Itgae^tm1Cmp^*/J, (KO) were purchased from the Jackson Laboratory (Bar Harbor, ME). Colonies of WT and KO mice were expanded from breeder stocks and maintained in house. CBy.Cg-*Thy1^a^* Tg(TcraCl1,TcrbCl1)1Shrm/J (BALB/c-Thy1.1) mice were kindly provided by Dr. Donna Farber. Thy1.1^+^Thy1.2^+^ CD103-deficient mice were generated by interbreeding BALB/c-Thy1.1 and KO (Th1.2^+^) mice, followed by backcrossing of the F1 generation to the KO parental strain to generate Thy1.1^+^Thy1.2^+^ KO mice, identified by surface staining using mAbs to Thy1.1, Thy1.2 and CD103. All mice were housed in specific pathogen-free conditions in the animal facilities at the University of Maryland, Baltimore, MD and The Ohio State University, Columbus, OH. Animal studies were approved by University Laboratory Animal Resources at The Ohio State University (approval 2009A0213) and the Office of Animal Welfare Assurance at the University of Maryland, Baltimore (approval 11-19-04).

### Antibodies

Directly conjugated anti-mouse antibodies used included CD3-FITC, CD8-FITC, CD8-PerCP, CD8-APC, Thy1.1-PerCP, Thy1.2-PE, M290 (anti-CD103)-PE and FITC, IFN-γ-PE, TNF-α-PE, IL-2 and isotype-matched controls, all purchased from BD Biosciences Pharmingen (San Diego, CA).

### GVHD and GVT models

For CD8 T cell transfer experiments, lethally-irradiated (9 Gy, ^137^Cs 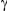-radiation, split into 2 doses) A/J hosts (H-2^a^) were reconstituted within 4-6 hours with a single 0.5 ml intravenous inoculum containing 1.0×10^7^ BALB/c-WT bone marrow cells (BMC) and 1.0×10^7^ naïve or alloantigen-primed CD8 T cells from either BALB/c-WT or BALB/c-KO mice. Donor splenocytes (SC) were enriched for CD8 T cells by treatment with mAbs to CD4 (RL172.4), heat stable antigen (J11D) and I-E^d^ (14.4.4s), followed by incubation in 1/10 Low-Tox M rabbit complement (Accurate Chemical and Scientific, Westbury, NY). The resulting cell suspensions contained <1% CD4^+^ T cells. In some experiments, recipient mice were also injected subcutaneously on the back with 1.0×10^6^ A/J background fibroblast sarcoma (SaI/N) tumor cells (kindly provided by Dr. Suzanne Ostrand-Rosenberg). To determine the role of CD103 in CD8 T cell accumulation in host tissue compartments, 2×10^6^ BALB/c-WT (Thy1.2^+^) and 2×10^6^ BALB/c-KO CD8 cells (Thy1.1^+^Thy1.2^+^) were transferred in combination with BALB/c-WT BMCs (Thy1.2) into lethally-irradiated A/J hosts. Mice were randomly grouped before and after irradiation. Control irradiated untreated mice were also included in each experiment.In some experiments, GVHD pathology was prevented by transfer of 1.0×10^7^ alloantigen-primed WT or KO CD8 T cells into syngeneic BALB.scid mice together with SaI/N cells.

Host survival following alloSCT was monitored daily, and GVHD severity was quantitated as described by Cooke et al. [18]. Briefly, recipient mice were scored for five parameters (weight loss, skin integrity, fur texture, mobility, and posture) using a scale of 0–2 (0 = normal, 1 = mildly abnormal, and 2 = severely abnormal). The GVHD severity score was the sum of the scores for each individual criterion. Death due to GVHD was defined by the absence of tumor and the presence of clinical signs of GVHD. GVT effects were assessed by monitoring tumor size every 2 days. Death due to tumor was defined by the absence of GVHD and the presence of measurable tumor. For histologic analyses, host organs were harvested at the designated times, fixed in 10% phosphate-buffered formalin, and embedded in paraffin. Sections (6 µm) were stained with hematoxylin and eosin.

### Flow cytometry

Lymphocytes infiltrating the intestinal epithelium and LP during GVHD were isolated as previously described [7]. Lymphocytes were isolated from lymphoid organs by mincing with forceps and passage through nylon mesh of 100-µm pore size. Liver-infiltrating lymphocytes were harvested as previously described [19]. To isolate tumor-infiltrating lymphocytes, the tumor and the surrounding tissue within 1 mm were minced and the tumor pieces were incubated with stirring at 37°C for 30 min in a mixture of collagenase (Worthington Biochemical, Freehold, NJ), soybean trypsin inhibitor (Sigma-Aldrich, St. Louis, MO), and DNase (Roche, Indianapolis, IN). Host organ- and tumor-infiltrating lymphocytes were isolated by centrifugation on Lympholyte-M (Cedarlane Laboratories, Hornby, Ontario, Canada). Lymphocyte populations were washed twice in FACS buffer prior to antibody staining. Lymphocyte populations were stained and analyzed as previously described [7]. Assays were performed according to the manufacturer's protocol.

### In vivo cytotoxicity assay

A modification of the technique originally described by Oehen and Brduscha-Riem [20] was adapted for use in these studies. In brief, at day 7 after alloSCT, A/J recipients of CD8 T cells from WT or KO donors were injected by tail vein with 2.0×10^7^ SC from WT donors (donor-type) and 2.0×10^7^ SC from A/J donors (host-type). SC were labeled with 0.5 µM or 5.0 mM CFSE (Invitrogen Corp.), respectively, prior to transfer to generate cells distinguishable by flow cytometry. Eighteen hours later, animals were bled and erythrocytes were lysed using red blood cell lysing buffer (Sigma). The proportions of CFSE-labeled host (low intensity) and donor cells (high intensity) were determined by flow cytometry (BD Biosciences Pharmingen, San Diego, CA) and an in vivo cytotoxicity index (measuring the in vivo cytotoxic response to host-type targets) was computed as the percentage of remaining host-type cells divided by the percentage of remaining donor-type cells.

### Statistical analyses

To assess statistical significance, Student's *t* tests and paired *t* tests were performed using SigmaPlot 2000 software (SPSS Inc., Chicago, Illinois). The Kaplan-Meier product limit method was used to obtain the survival probability and the log-rank test was applied to compare the survival curves. P<0.05 was considered statistically significant.

## Supporting Information

Figure S1 **CD103 expression by CD8 T cells in the host intestine (A) and spleen (B).** CD8 T cells primed to A/J alloantigens from either BALB/c-WT (WT) or BALB/c-CD103 KO donors were adoptively transferred into lethally irradiated A/J recipient mice in combination with WT BMC. Data shown are 2-dimensional plots of CD103 expression vs. CD8 expression for gated CD8+ lymphocytes in the two compartments.
(TIF)

Figure S2 **CD103 expression is not required for CD8-mediated cytotoxicity to host cells during GVHD.** Lethally-irradiated A/J mice received BMC plus alloantigen-primed splenic CD8 T cells from either BALB/c-WT (WT) or BALB/c-CD103 KO donors. At day 7 post-transplant recipients mice received a mixture of WT (CFSE^lo^) and A/J (CFSE^hi^) splenocytes i,v,; 18 hrs later mice were bled and in vivo cytotoxicity indices calculated. Data shown are cytotoxicity indices in recipients of CD8 T cells from WT (n = 4) or KO (n = 4) donors (mean ± SEM).
(TIF)
