# Supplementary figures and images for: CD103 Deficiency Prevents Graft-versus-Host Disease but Spares Graft-versus-Tumor Effects Mediated by Alloreactive CD8 T Cells
Source: PLoS One. 2011 Jul 14;6(7):e21968. doi: 10.1371/journal.pone.0021968 (PMC3136479; doi:10.1371/journal.pone.0021968)

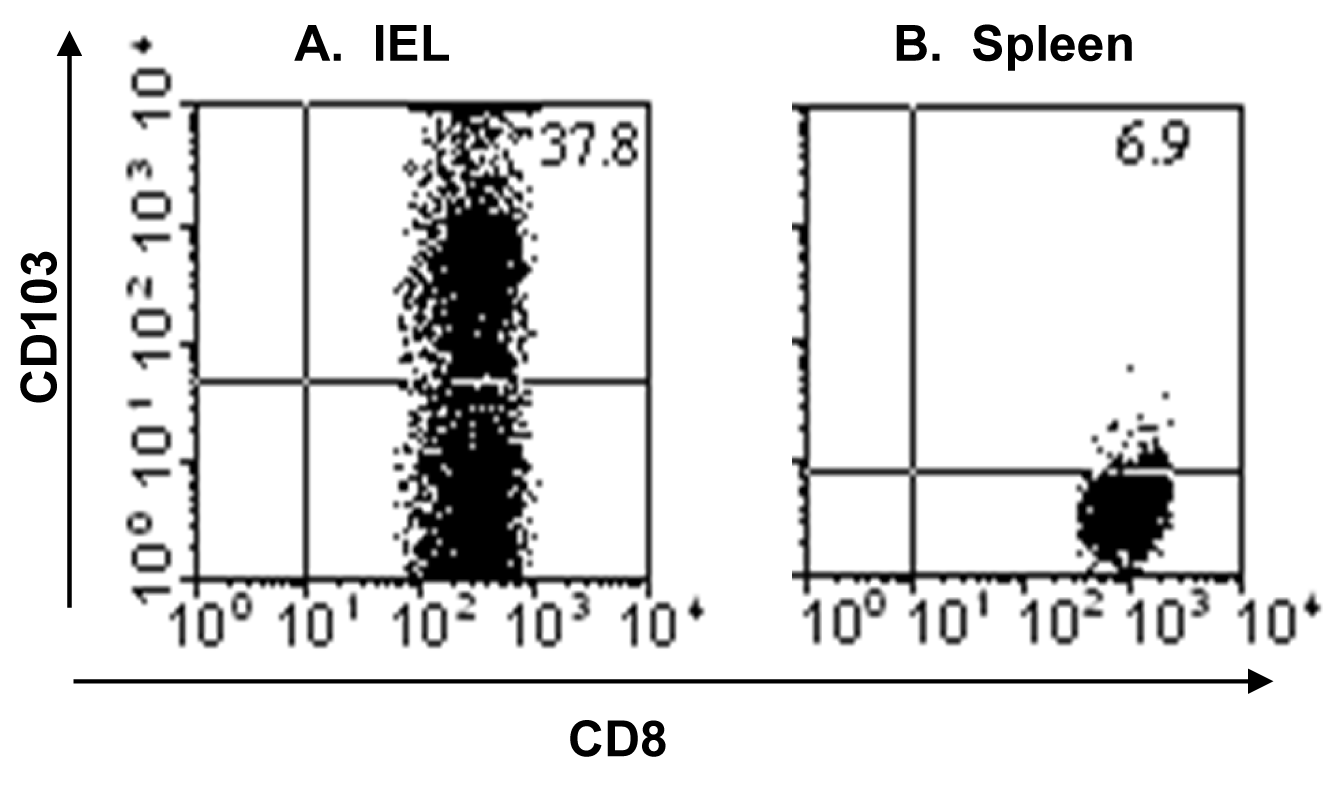

Supplement: Figure S1 — CD103 expression by CD8 T cells in the host intestine (A) and spleen (B). CD8 T cells primed to A/J alloantigens from either BALB/c-WT (WT) or BALB/c-CD103 KO donors were adoptively transferred into lethally irradiated A/J recipient mice in combination with WT BMC. Data shown are 2-dimensional plots of CD103 expression vs. CD8 expression for gated CD8+ lymphocytes in the two compartments. (TIF) [file pone.0021968.s001.tif]

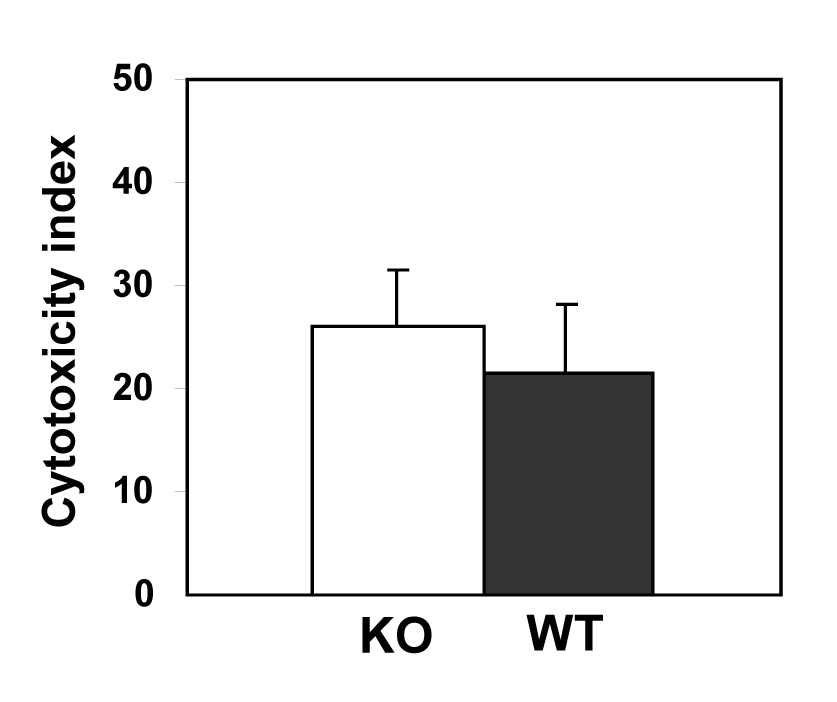

Supplement: Figure S2 — CD103 expression is not required for CD8-mediated cytotoxicity to host cells during GVHD. Lethally-irradiated A/J mice received BMC plus alloantigen-primed splenic CD8 T cells from either BALB/c-WT (WT) or BALB/c-CD103 KO donors. At day 7 post-transplant recipients mice received a mixture of WT (CFSElo) and A/J (CFSEhi) splenocytes i,v,; 18 hrs later mice were bled and in vivo cytotoxicity indices calculated. Data shown are cytotoxicity indices in recipients of CD8 T cells from WT (n = 4) or KO (n = 4) donors (mean ± SEM). (TIF) [file pone.0021968.s002.tif]
